# Landscape of Congenital Adrenal Hyperplasia Newborn Screening in the United States

**DOI:** 10.3390/ijns6030064

**Published:** 2020-08-14

**Authors:** Sari Edelman, Hiral Desai, Trey Pigg, Careema Yusuf, Jelili Ojodu

**Affiliations:** Association of Public Health Laboratories, Silver Spring, MD 20910, USA; hiral.desai@aphl.org (H.D.); trey.pigg@aphl.org (T.P.); careema.yusuf@aphl.org (C.Y.); jelili.ojodu@aphl.org (J.O.)

**Keywords:** newborn screening, congenital adrenal hyperplasia, NewSTEPs

## Abstract

Newborn screening (NBS) is a state-based public health program that aims to identify newborns at risk of certain disorders in the first days after birth to prevent permanent disability or death. Disorders on the Health and Human Services Federal Advisory Committee’s Recommended Uniform Screening Panel (RUSP) have been adopted by most state NBS programs; however, each state mandates specific disorders to be screened and implements their own system processes. Congenital adrenal hyperplasia (CAH) was added to the RUSP in 2005, and currently all 53 NBS programs universally screen for it. This paper provides a landscape of CAH screening in the United States, utilizing data voluntarily entered by state NBS programs in the Newborn Screening Technical assistance and Evaluation Program data repository. Data reported encompasses NBS state profile data (follow-up, disorder testing and the reporting of processes and methodologies for screening), quality indicator data (timeliness of CAH NBS) and confirmed cases. This comprehensive landscape analysis compares the CAH NBS systems across the US. This is vital in ultimately ensuring that newborns with CAH at risk of salt crisis receive appropriate intervention in a timely manner.

## 1. Introduction

Newborn screening (NBS) is a state-based public health program that aims to identify newborns at risk for certain disorders in the first days or weeks after birth, in order to prevent permanent disability or death. The US Health and Human Services (HHS) Federal Advisory Committee on Heritable Disorders in Newborns and Children (ACHDNC) evaluates and recommends disorders to be included in the Recommended Uniform Screening Panel (RUSP). However, each state mandates the specific disorders to be screened by their own program, implements system processes including the follow-up of out-of-range (screen-positive) results, and is responsible for the quality improvement and assurance of the entire NBS system [1].

This paper provides a landscape of congenital adrenal hyperplasia (CAH) screening in the United States, utilizing data voluntarily entered by state NBS programs in the Newborn Screening Technical Assistance and Evaluation Program (NewSTEPs) data repository.

**Figure 1:**
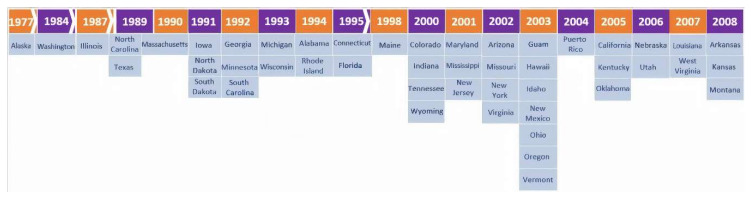
Timeline of congenital adrenal hyperplasia (CAH) newborn screening implementation in the United States.

By the time CAH was added to the RUSP in 2005, 72% (*n* = 38/53) of NBS programs were already universally screening for the disorder. Of NBS programs, 19% (*n* = 10/53) implemented universal screening for CAH in 2005 or after, and 9% (*n* = 5/53) of NBS programs did not know or did not have access to that year of implementation information. Currently, all 53 NBS programs universally screen for CAH, programs which consist of the 50 US states, the District of Columbia, Puerto Rico and Guam (Figure 1). CAH, caused by steroid 21-hydroxylase deficiency, occurs in 1/15,000 births, and is more common in certain populations [2]. NewSTEPs collects and classifies CAH cases in three categories: Classic 21-hydroxylase deficiency (salt-wasting), Classic 21-hydroxylase deficiency (simple virilizing), and other adrenal disorder. By identifying babies with severe, salt-wasting CAH before they develop adrenal crises, screening reduces morbidity and mortality, especially among affected boys. Diagnosis is based on elevated levels of 17-hydroxyprogesterone (17-OHP), the preferred substrate for steroid 21-hydroxylase [3].

## 2. Materials and Methods

### 2.1. NewSTEPs State Profiles

NewSTEPs [4], a program of the Association of Public Health Laboratories (APHL), is the national NBS resource center designed to provide data, technical assistance and training to US NBS programs and assist them with quality improvement initiatives. It functions with the goal of improving outcomes for newborns by facilitating NBS initiatives and programmatic outcomes, including the offering of expertise in NBS program development and evaluation, member connection and data analysis to improve the overall quality of the NBS system [5]. Aligning with this goal, NewSTEPs maintains a centralized and secure data repository that is designed to collect comprehensive data on NBS programs. The data collected encompass NBS state profile data (public facing, programmatic overview data), quality indicator data (restricted to authorized users, metrics of program performance) and confirmed cases (restricted to authorized users). NewSTEPs collects individual-level confirmed case data, as well as aggregate counts of confirmed cases by NBS programs. While registration for the repository is open to all who are interested, restricted data elements, which include confirmed cases and quality indicators, are only accessible to individuals who are granted data access and data entry permissions by each NBS program. NewSTEPs requires that NBS programs have a signed Memorandum of Understanding (MOU) with APHL in order to enter restricted data elements. This manuscript includes state profile data from all 53 NBS programs in the US, as well as individual-level case data from 35 NBS programs with signed MOUs. This manuscript refers to non-CAH cases as all cases from the RUSP, excluding CAH, which have been entered into the NewSTEPs data repository.

NewSTEPs collects data in accordance with an established data entry timeline [6]. NBS programs are encouraged to update state profile and quality indicator data on an annual basis, and case data from two years prior on an annual basis. This is to accommodate for the time it takes to resolve and close out cases. Individual-level CAH cases adhere to a set of public health surveillance case definitions that have been developed by the NBS community to facilitate common classifications for diagnoses across programs (see Supplementary Materials for NewSTEPs’ CAH case definition). Consensus public health surveillance case definitions for NBS disorders allow for the consistent categorization and tracking of newborns identified with disorders [7].

### 2.2. CAH Data Request

NewSTEPs followed the formal policies and procedures established for requesting case-level data for use in this paper. The request for data was channeled through APHL’s governance structure and directed to the Data Review Workgroup, which is charged with providing expertise in order to make recommendations to NewSTEPs staff and the NewSTEPs Steering Committee on any requests made for data collected within the repository. The Data Review Workgroup has no objections to the use of data presented in this paper.

State profile data are represented as of March 2020. In total, 53 NBS programs contribute state profile data, however not all programs contribute data to all fields. NBS programs reporting individual case data (*N* = 35) are represented from 2015 to 2017 to align with the NewSTEPs’ data entry timeline. 

### 2.3. CAH Data Query and Analytics 

In addition to routine data submission efforts (customized technical assistance, interactive Tableau data visualizations, phone and email reminders of data entry timeline, customized tutorials of the NewSTEPs website and data repository, user guides and reports of frequently asked questions, and import templates and other tools to facilitate data entry), NBS program-specific encrypted CSV files were sent to NBS program case data entry staff with instruction to review fields to ease the data submission of requested missing fields of cases entered. The analyzed CAH case data fields in this report included gestational age, birth weight, biological sex, societal sex, family history, final diagnosis, mutational analysis, 17-OHP serum levels and timeliness intervals for CAH screening. The number of missing fields were specific to each program. These fields were selected as they offered the most quantitative and qualitative data to allow for a sufficient landscape of CAH screening.

These efforts were followed up with targeted phone outreach. Efforts were successful in cleaning data and updating missing fields, however not all missing fields were updated due to programs not collecting or not having certain information. Data were queried from the repository using a combination of Structure Query Language (SQL) and Tableau. Open source libraries and statistical functions from R 3.6.3 were utilized to analyze data and explore dataset patterns by parsing elements queried from the NewSTEPs data repository.

## 3. Results

### 3.1. State Profile Data

One screen vs. two screen: Each state or territory has mandates to screen newborns, and these mandates specify if newborns will receive one or two screens. Thirteen states (Alabama, Arizona, Colorado, Delaware, Idaho, Maryland, Nevada, New Mexico, Oregon, Texas, Utah, Washington and Wyoming) are considered two-screen states, because they require that a second dried blood spot (DBS) specimen be collected on all newborns regardless of the results of the first newborn screen. Newborns in the other 40 states and territories typically undergo a single newborn screen. If a specimen is collected too early, or if there is an unsatisfactory specimen due to collection or transport errors, an additional screen may be prompted [1].

Short-term follow-up: Of the NBS Programs, 62% (*n* = 33) define short-term follow-up in their state as until a diagnosis is made or ruled out; 21% (*n* = 11) define it as until the baby is on treatment; 4% (*n* = 2) define it as until confirmatory testing is performed; 13% (*n* = 7) define it as other. Other responses included a combination of until diagnosis is made or ruled out, or the baby is on treatment (*n* = 3); a combination of until confirmatory testing is performed, or the baby has seen a specialist, is on treatment or the diagnosis is made or ruled out (*n* = 3); and a breakdown of short-term follow-up of borderline (until diagnosis is made and ruled out, or three months) versus presumptive positive (until diagnosis is made and ruled out, or one year) (*n* = 1). Although the Clinical and Laboratory Standards Institute (CLSI) does not define short-term follow-up specifically, follow-up is defined as actions taken to ensure that a person whose test results are screen positive or invalid receives appropriate further tests and evaluation in a timely fashion, and as actions taken that ensure the newborn screening system evaluates the effectiveness of screening [8].

**Figure 2:**
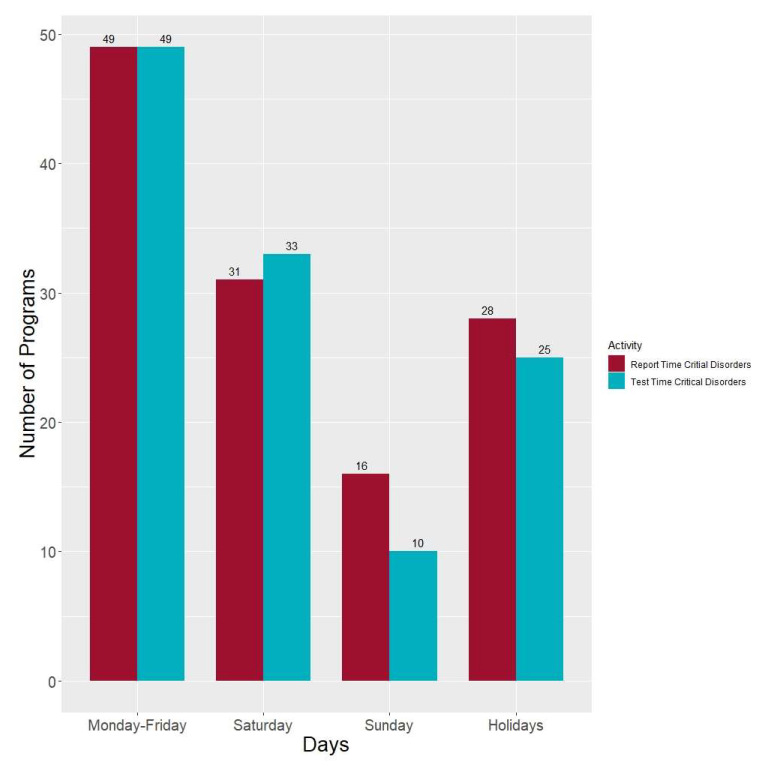
Days newborn screening (NBS) laboratories test for time-critical disorders and days NBS offer follow-up reports on time-critical disorders (*N* = 49).

Time-critical disorder screening and reporting out processes: CAH is considered a time-critical disorder [9]. One hundred percent of NBS programs that provided data (*n* = 49/49) run tests for time-critical disorders, inclusive of CAH testing, five days of the week on Monday through Friday. Sixty-seven percent (*n* = 33/49) run tests for time-critical disorders on Saturday, 20% (*n* = 10/49) on Sunday, and 51% (*n* = 25/49) during holidays (Figure 2). All of the NBS programs (*n* = 49/49) report on time-critical disorders Monday to Friday. Sixty-three percent (*n* = 31/49) carry out tests on Saturday; 33% (*n* = 16/49) carry out tests on Sunday; and, 57% (*n* = 28/49) carry out tests on holidays (Figure 2). 

### 3.2. Methodology for Screening 

All 53 NBS programs screen for CAH using fully integrated fluoroimmunoassay (FIA) as their first screen method, with 17-OHP as the target. Twenty-nine programs use the PerkinElmer Genetic Screening Processor (GSP) for first tier screening, while the remaining 24 programs use the PerkinElmer AutoDELFIA instrument. Both instruments apply the same immunoreaction technology and have the same reagents to measure 17-OHP, however the GSP is a newer model with higher throughput. 

Eleven NBS programs provided first-screen, second tier methodology and target data for CAH. Six NBS programs reported also using FIA for their second tier, andfive NBS programs extracted 17-OHP specifically. Four NBS programs reported using liquid chromatography tandem mass spectrometry (LC-MS/MS) for their second tier, with a combination of 17-OHP (*n* = 4), adrostenedione (*n* = 2), cortisol (*n* = 2), 17-OHP + adrostenedione/cortisol (*n* = 2), 11-Deoxycorticosterone (*n* = 1) and 21-Deoxycortisol (*n* = 2) as the screening target (programs can enter multiple screening targets). The remaining program reported sending specimens for second tier screening to Mayo Clinic Laboratories.

### 3.3. Confirmed Case Data

NewSTEPs collects individual-level confirmed cases in the NewSTEPs data repository in accordance with public health surveillance case definitions, as well as aggregate case counts per condition. From the years 2015 to 2017, 495 individual-level cases were entered by 35 participating NBS programs. Comparatively, 10,892 individual non-CAH cases were entered by 36 participating NBS programs. Furthermore, 826 aggregate case counts of CAH were entered from 49 participating NBS programs within the same timeframe.

**Figure 3:**
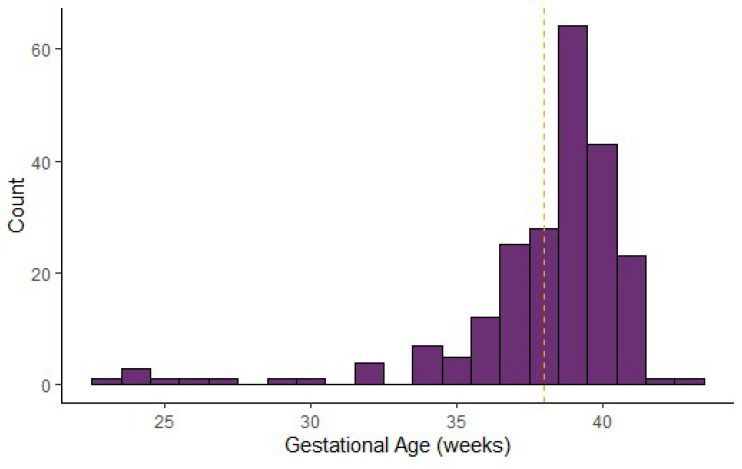
Congenital adrenal hyperplasia cases: distribution of gestational age (*N* = 222).

**Figure 4:**
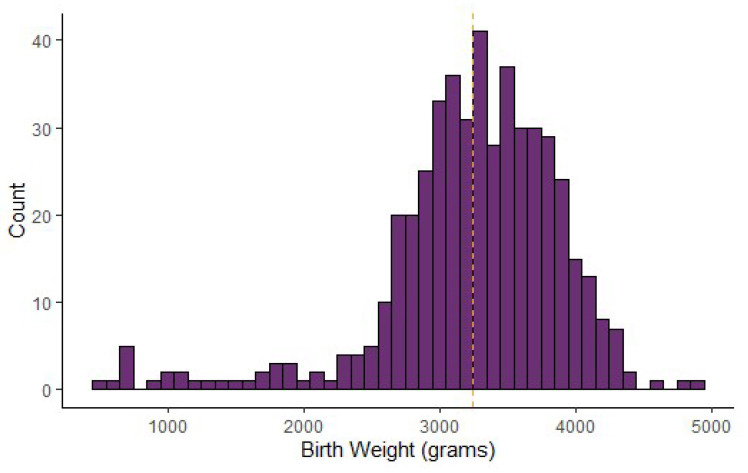
Congenital adrenal hyperplasia cases: distribution of birth weight (*N* = 484).

Gestational age and birth weight: The median gestational age of individual-level CAH cases entered (*N* = 222) was 39 weeks, with an interquartile range (IQR) of 37–40 weeks. This was the same as the median gestational age of all non-CAH cases entered in the data repository (*N* = 6531). The median birth weight of CAH cases entered (*N* = 222) was 3317.5 g with an IQR of 2976.5–3700 g. Similarly, the median birth weight of all non-CAH cases entered in the data repository was 3180 grams (*N* = 10,015). Figure 3 and Figure 4 show the distribution of gestational age and birth weight. The median birth weight for 262 male infants was 3371.5, with an IQR of 2975.5–3757.25 grams; the median birth weight for 203 females was 3270 with an IQR of 2955–3577.5 grams. The p-value of 0.03 indicates that the differences in the distribution of male and female birth weight are statistically significant.

### 3.4. Diagnostic Workup Data

Final diagnosis: Of these cases, 37% (*n* = 185/495) had a final diagnosis of Classic 21-hydroxylase deficiency (salt-wasting); 6% (*n* = 31/495) had a final diagnosis of Classic 21-hydroxylase deficiency (simple virilizing); 6% (*n* = 27/495) had a final diagnosis of other adrenal disorder, and 51% (*n* = 252/495) did not provide sufficient data to classify the type of CAH. The distribution of phenotypes by final diagnosis did not vary considerably year to year. 

**Figure 5:**
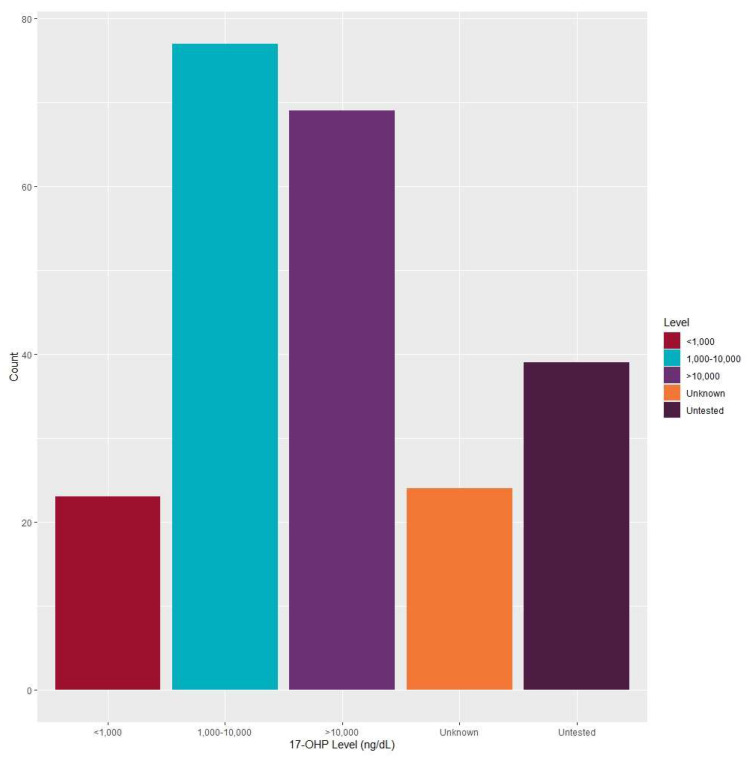
Congenital adrenal hyperplasia cases: 17-OHP serum levels (*N* = 232).

17-OHP Serum Levels: The 17-OHP serum level ranges can be observed in Figure 5 from a total of 232 entered cases, and include levels reported as “unknown” or “untested.” Data was not reported for 263 cases. Of the 232 cases entered, 33.2% (*n* = 77) of 17-OHP serum levels were between 1000 and 10,000 ng/dL, 29.7% (*n* = 69) were greater than 10,000 ng/dL, and 9.9% (*n* = 23) were less than 1000 ng/dL (Figure 5).

According to the American College of Obstetricians and Gynecologists and the Society for Maternal Fetal Medicine, a pregnancy is considered early term at 37 weeks of gestation through 38 6/7 weeks of gestation, and full term at 39 weeks of gestation through 40 6/7 weeks of gestation [10]. Of the 77 cases that had 17-OHP levels between 1000 and 10,000 ng/dL, the gestational age was between 25 and 38 weeks for 24 infants, 39 and 40 weeks for 35 infants, it was 41 weeks for 8 infants and was unreported for 10 infants. One case indicated a 17-OHP level less than 1000 ng/dL, but reported an actual value of 627 ng/dL with a final diagnosis of Classic 21-hydroxylase deficiency (salt-wasting). The gestational age for this infant was 39 weeks. Of the 23 cases that reported 17-OHP levels less than 1000 ng/dL, 6 infants’ gestational ages were between 32 and 38 weeks, 7 infants’ gestational ages were between 39 and 40 weeks, 3 infants’ gestational ages were between 41 and 43 weeks, and data was not reported for 7 infants.

Biological and societal sex: According to NewSTEPs, biological sex is determined at the birthing facility and included on the dried blood spot card, while societal sex is determined during the diagnostic process. Of the 203 cases entered with a female biological sex, 33% (*n* = 67) also had a female societal sex and 67% (*n* = 136) had an unknown or unspecified societal sex, or did not provide data on societal sex. Of the 263 cases entered with a male biological sex, 38% (*n* = 101) also had a male societal sex, 0.4% (*n* = 1) had a female societal sex and 61% (*n* = 161) had an unknown or unspecified societal sex, or did not provide data on societal sex. Of the 11 cases entered with no data on biological sex, 9% (*n* = 1) had a male societal sex, and 91% (*n* = 10) did not provide data on societal sex. Of the 18 cases entered with unknown or unspecified biological sex, 33% (*n* = 6) had a female societal sex, 6% (*n* = 1) had a male societal sex and 61% (*n* = 11) had an unknown or unspecified societal sex, or did not provide data on societal sex.

**Figure 6:**
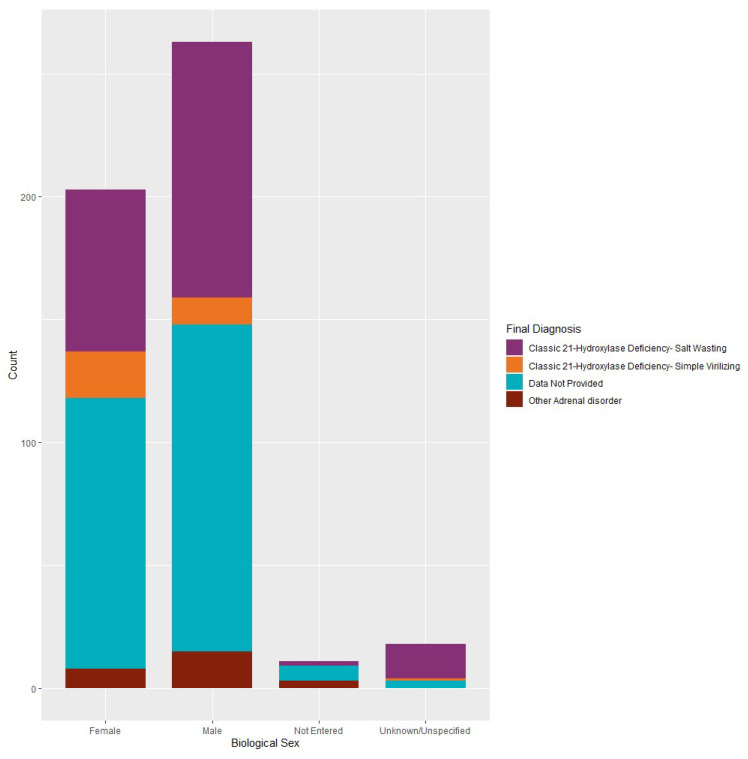
Congenital adrenal hyperplasia cases: final diagnosis by biological sex from 2015 to 2017 (*N* = 495).

Out of the cases that did report biological sex, Classic 21-hydroxylase deficiency (salt-wasting) was the most common final diagnosis amongst females (*n* = 66), males (*n* = 104) and infants with unknown or unspecified biological sex (*n* = 14), whereas another adrenal disorder was the most common final diagnosis amongst those cases for whom data was provided on biological sex (*n* = 4; Figure 6). 

### 3.5. Timeliness Data

**Figure 7:**
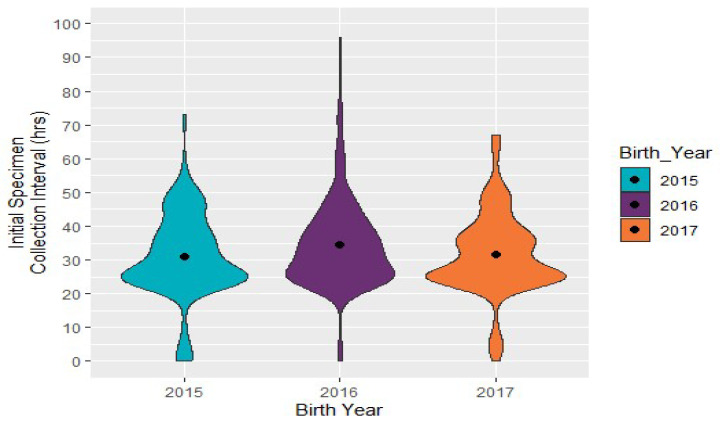
Congenital adrenal hyperplasia cases: time from birth to specimen collection in hours (*N* = 478).

Initial specimen collection: The recommendations for initial specimen collection are between 24 and 48 h after birth in every state NBS program, except for California [11]. The median time for specimen collection (*N* = 482) between 2015 and 2017 was 31 h, with an IQR of 24.5–39 h (Figure 7). A one-sample *t*-test was used, and showed a p-value of 6.7 × 10^−7^, which is less than the significance level of 0.05. The plot below visualizes density, indicating that most specimens were collected between 24 and 30 h of birth. Specimen collection time did not vary considerably from 2015 to 2017.

Specimen receipt interval: The median time from birth to receipt of the specimens by the laboratory was 3 days, with an IQR of 3–4 days (*N* = 470). The specimen receipt interval remained similar from 2015 to 2017. The *p*-value of 0.17 indicates that there is no significant change in specimen receipt interval through the years.

**Figure 8:**
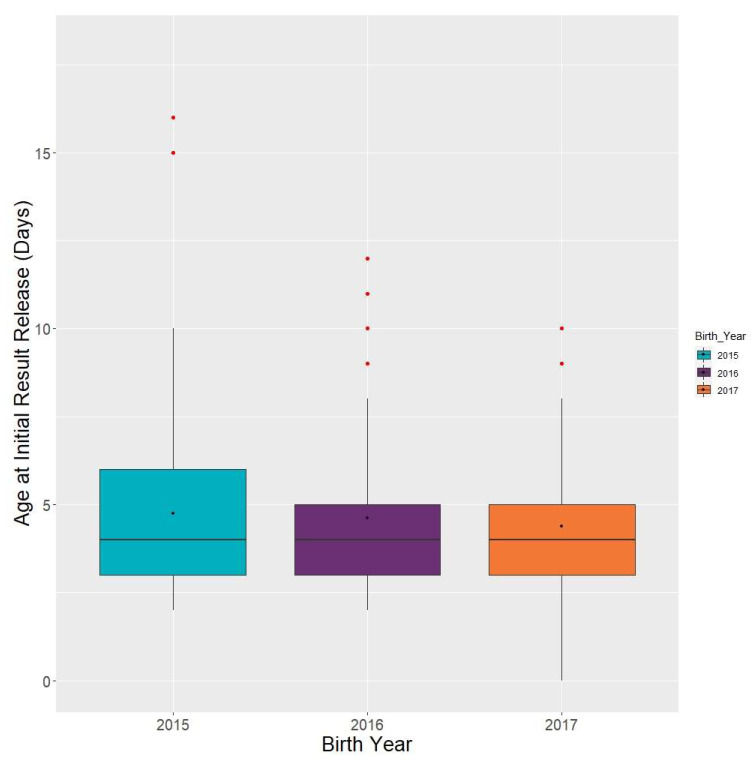
Congenital adrenal hyperplasia cases: time interval from birth to release of out-of-range results (*N* = 453).

Initial specimen result release: The median time from birth to release of out-of-range CAH results (*N* = 453) was 4 days, with an IQR of 3–6 days (Figure 8). Comparatively, the median release of out-of-range non-CAH results (*N* = 9564) from birth was 6 days. The *p*-value of 0.12 indicates that there was no significant reduciton in initial result release.

### 3.6. Subsequent Specimen Collection

**Figure 9:**
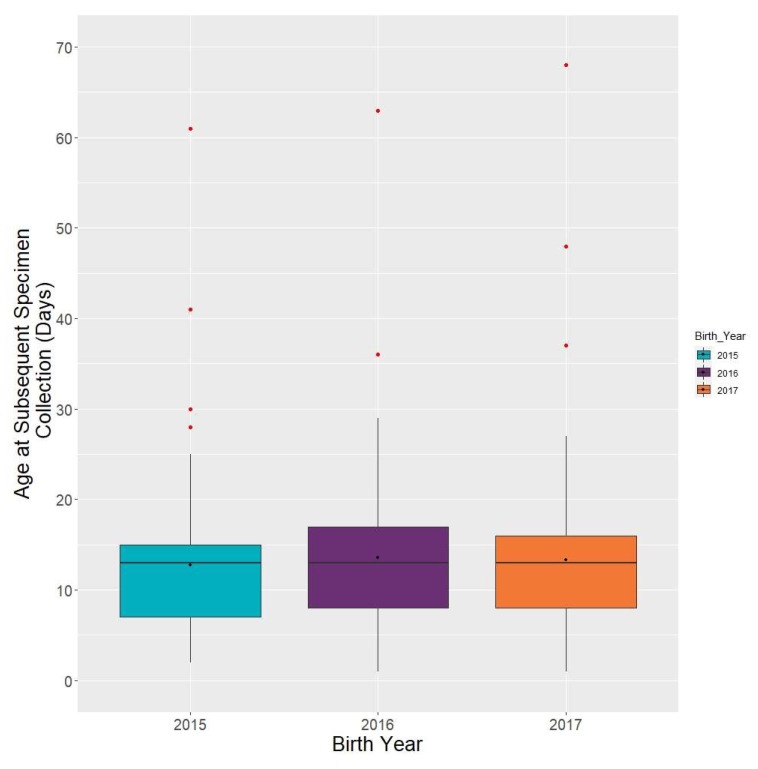
Congenital adrenal hyperplasia cases: age at subsequent specimen collection (*N* = 287).

Subsequent specimen collection: NewSTEPs defines a subsequent specimen as any specimen received at the laboratory for a given newborn screen after the first specimen has been received. A subsequent specimen may be requested based on a borderline result from the first specimen, or an unacceptable first specimen. There may be multiple subsequent specimens per screen [12]. The median for subsequent specimen collection (*N* = 287) was 13 days of life, with an IQR of 8–16 days. (Figure 9). The *p*-value of 0.74 indicated that subsequent specimen collection through the years has not significantly changed.

Subsequent specimen receipt interval: The median time from birth to subsequent specimen receipt at laboratory (*N* = 287) was 16.1 days, with an IQR of 10–20 days. The *p*-value of 0.44 indicated that there was no real change in subsequent specimen receipt interval from 2015 to 2017. 

**Figure 10:**
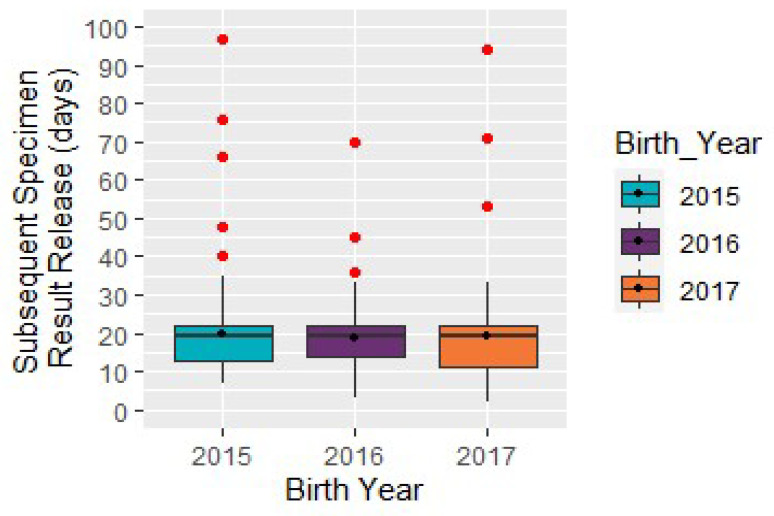
Congenital adrenal hyperplasia cases: subsequent specimen result release of out-of-range results, by year (*N* = 287).

Subsequent specimen receipt result release interval: The median age of the infant at subsequent specimen receipt of out-of-range CAH results was 19 days (*N* = 287; Figure 10). The *p*-value 0.85 showed that there was not a significant change in the result release interval from 2015 to 2017. In comparison, the median time interval of the subsequent specimen release of out-of-range non-CAH results (*N* = 3877) from birth was 17 days.

**Figure 11:**
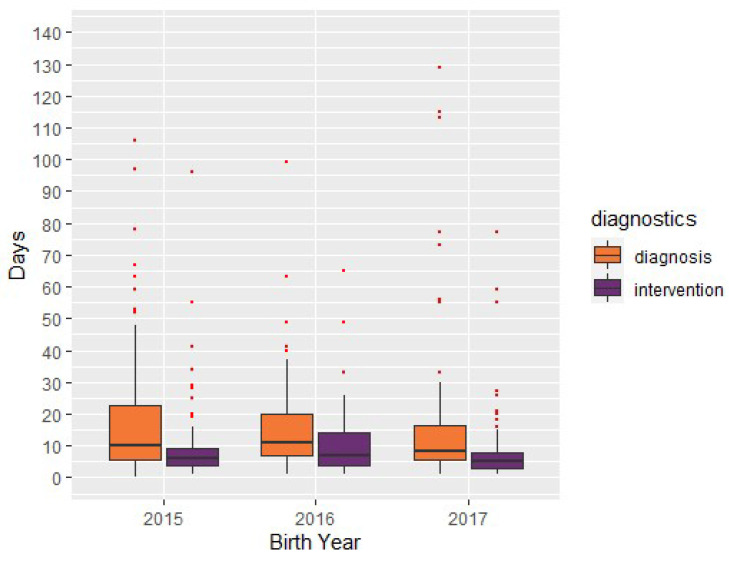
Congenital adrenal hyperplasia cases: time interval from birth to intervention and confirmation of diagnosis, by year.

NewSTEPs defines intervention by an appropriate medical provider as the date of the clinic visit or hospital consultation to evaluate the potential diagnosis of CAH [13]. The median time from birth to intervention by an appropriate medical provider for CAH cases (*N* = 233) was 6 days. The median time from birth to intervention for all non-CAH cases (*N* = 6713) was 13 days. The median time from birth to confirmation of diagnosis of CAH cases (*N* = 243) was 10 days (Figure 11). The p-values of 0.12 and 0.31 indicated that there were no significant changes in the time to intervention or diagnosis, respectively, for CAH cases screened from 2015 to 2017. The median time from birth to confirmation of diagnosis was also 10 days for the CAH cases submitted by the 13 NBS programs who entered data for all three years. Comparatively, the median time from birth to confirmation of diagnosis for all non-CAH cases (*N* = 7505) was 24 days. 

## 4. Discussion

### 4.1. State Profile Data

While most NBS programs are considered one-screen states, there are certain circumstances, such as if a specimen is collected too early or if there is an unsatisfactory specimen due to collection or transport errors, that may prompt an additional screen. A 2015 study indicated that there is no clear consensus among state NBS programs on whether the routine second screening of newborns can identify clinically relevant cases of CAH [14].

Although NBS programs define short-term follow-up differently, every program conducts follow-ups on infants at risk of having an NBS disorder. Most programs define short-term follow-up in their state as until a diagnosis is made or ruled out. Furthermore, NBS programs differ in hours of operation depending on day of the week. Certain activities may be prioritized or not conducted at all, depending on state-specific standard operating procedures. However, all NBS programs run tests for and report on results for time-critical disorders Monday to Friday. It should be noted that 16 NBS programs have laboratories that are open five days a week, 26 are open six days a week, and 11 are open seven days a week. A recent publication found that laboratory operating hours are a critical factor associated with timeliness, and that infants receiving services independent of the day of the week they were born will show a reduced risk of tragic outcomes for their individual families [15].

All NBS programs screen for CAH using FIA as their first screen method, with 17-OHP as the target. About half of the programs use the PerkinElmer GSP, and the other half use the PerkinElmer AutoDELFIA instrument. For the second tier, about half of the programs reporting data use FIA, and the other use LC-MS/MS with varying method targets.

### 4.2. Confirmed Case Data

NBS programs report the number of aggregate cases of each disorder on the RUSP identified by DBS NBS to the NewSTEPs data repository. For the years 2015, 2016 and 2017, there has been a total of 826 aggregate case counts of CAH from 49 participating NBS programs, as of March 2020.

NBS programs also report confirmed infant-level case data to the NewSTEPs data repository. From the years 2015–2017, 495 individual cases were entered in the data repository from 35 participating NBS programs. It should be noted that state participation per year varied. For example, the number of state NBS programs contributing data are different in 2015 than in 2016 and 2017. There are several reasons for this discrepancy between aggregate and individual cases in the NewSTEPs data repository. First, not all NBS programs are able to provide all required fields for individual case entry, specifically diagnostic workup data. NBS programs may not have received this information back from physicians or specialists, or may have restrictions on the level of data they can report to NewSTEPs and outside organizations. Furthermore, NBS programs may not collect the level of data that NewSTEPs requests, such as some of the demographic fields (gestational age, and biological and societal sex). Barriers to data entry include data entry staff shortages, high turnovers of staff and a lack of NewSTEPs integration into workflows. Although NewSTEPs adheres to a data entry timeline, performs numerous methods of targeted outreach to state NBS programs for data collection and strives to ease and automate this process, all data entered in the repository is voluntary.

Almost all NBS programs were able to provide data on birth weight, however gestational age data proved more difficult, with 45% (*n* = 222/495) of all cases entered providing this data. Reasons reported for this include the following: no demographic data was available, previous laboratory information management systems did not capture this data, some cases were reported to the NBS program with missing data, and NBS programs are unable to report gestational age or do not collect gestational age. There was no difference in median gestational age or median birth weight among CAH cases and non-CAH cases, indicating that having CAH does not impact these factors.

Affected females with CAH may have ambiguous genitalia that alert medical professionals at the clinical level to their condition, and prompt appropriate diagnosis and treatment. In particular, affected females with Classic 21-hydroxylase deficiency (simple virilizing) are most often diagnosed immediately at birth, whereas affected males present with normal male genitalia, and thus their condition is detected at the prenatal or newborn screening level [16]. There was one instance in the repository in which societal sex differed from biological sex entered. There were eight additional instances in which no data was provided on biological sex, or it was unknown or unspecified, and societal sex was reported. It is possible that additional cases exist with incorrect initial sex assignation at birth. However, NewSTEPs does not collect long-term follow-up data, which is needed for this analysis. Of the information provided, in males, females and infants with unknown or unspecified biological sex, the final diagnosis was most commonly reported as Classic 21-hydroxylase deficiency (salt-wasting). This is in line with recent journal findings [17]. However, of the cases that did not provide data on biological sex, only one case had this as the final diagnosis; the remainder were identified as other adrenal disorders or data was not provided. Long-term follow-up data and complete diagnostic data are necessary for the further analysis of biological and societal sex among CAH cases. 

Despite the high null value for 17-OHP serum level reporting, most programs reported levels between 1000 and 10,000 ng/dL, followed by levels greater than 10,000 ng/dL. While types of CAH cases are categorized by NBS programs, NewSTEPs public health surveillance case definitions classify them into either definite, probable or possible. According to these definitions, 17-OHP serum levels between 1000 and 10,000 ng/dL have a greater likelihood of being probable cases of CAH, while serum levels greater than 10,000 have a greater likelihood of being definite cases [18]. There is a need for more complete clinical diagnostic information, particularly for those cases that do not fit within the typical reference range for CAH, in order to determine whether other interferences may have altered NBS values. Despite the high null value for phenotypes supplied by final diagnosis due to the aforementioned barriers, most individual cases entered into the NewSTEPs data repository had a final diagnosis of Classic 21-hydroxylase deficiency (salt-wasting). Final diagnoses of other adrenal disorders and Classic 21-hydroxylase deficiency (simple virilizing) had approximately the same numbers of cases entered. Studies have shown that gestational age is a better predictor of 17-OHP levels in newborns with CAH, and may guide the interpretation of screening results [19]. 

A recently published journal article noted the final status of CAH cases not being reported to state laboratories, and the need for quality improvement efforts to be directed at enhanced communication between clinicians and state laboratories, in addition to transparent reporting of states’ efforts on a national platform [17]. NewSTEPs data is in accordance with this finding, as NBS programs receive diagnoses back, but not necessarily detailed diagnostic level information. Other incomplete diagnostic workup fields, including mutation analysis data, further emphasize the need for more robust data collection. NewSTEPs does help with this effort and with quality improvement processes within state NBS programs.

### 4.3. Timeliness Data 

The median time of hours from birth to specimen collection for cases between 2015 and 2017 stayed within the 24–48 h collection timeframe recommended by the ACHDNC [11]. The median time of specimen collection did not vary considerably year to year, indicating that the NBS programs are performing well within this metric, although there is room for quality improvement. Thirty-three specimens were collected prior to 24 h of collection, which are deemed unsatisfactory specimens by NBS programs. Individual programs decide whether or not to screen these or wait for a subsequent specimen. 

Similarly, the median time of 4 days for CAH results being reported falls within the ACDHNC’s timeliness recommendation of time-critical results being reported within five days of life. This is slightly faster than the median time of 6 days for non-CAH results being reported, which also falls within ACHDNC’s timeliness recommendation of all results being reported within seven days of life. Furthermore, there were fewer results being reported after more than 5 days of life in 2017 and 2016 than in 2015. This highlights the significant efforts of NBS programs to operate their testing facilities and release out of range results as rapidly as possible. Quality improvement efforts implemented throughout NBS processes in order to sustain and improve upon timeliness metrics include the following: providing education to submitters and courier services about the importance of timely collection and shipment of specimens, expanding operating hours, improving laboratory workflows, and improving health information technology infrastructure in order to better transmit laboratory orders and results [15].

The median time for subsequent CAH results being reported was two days longer than the non-CAH results. This was an unexpected result, and further analysis is needed to determine cause.

There was no significant change in the median time interval from birth to receipt of specimens by the laboratory from 2015 to 2017. The reasons for outliers and delays in specimen receipt at the laboratory, as noted by NBS programs, included severe weather, lack of courier operations on holidays, delays in specimen shipment by midwives and the difficulty experienced by courier services in accessing rural or remote birthing facilities. Some of these challenges are being addressed by the aforementioned quality improvement efforts among NBS programs. NewSTEPs defines intervention by an appropriate medical provider as the earliest point at which a clinical action was rendered, based on the follow-up of the NBS results; this is inclusive of the date therapy was initiated or a decision was made to defer therapy based on current presentation. For CAH, it is the date of clinic visit or hospital consultation to evaluate the potential diagnosis of CAH:

- Standard confirmatory testing: Clinician draws electrolytes and 17-OHP;
- Electrolytes may or may not indicate need for urgent intervention, however a decision is rendered based on laboratory results and the clinical presentation of the infant;
- Advanced confirmatory testing: In cases in which exam or presentation strongly suggests diagnosis of CAH, additional adrenal testing may be warranted in consultation with endocrinologist.

The date of diagnosis is defined as when the diagnosis was confirmed upon elevated 17-OHP (+/− other abnormal adrenal hormone abnormalities) and evaluation by endocrinologist [13]. Fewer programs were able to report on intervention by medical provider and confirmation of diagnosis intervals. NBS programs often do not have this information, and may not receive this level of data back from clinicians. However, the median time to diagnosis and median time to intervention from data reported were not statistically significant, suggesting that all activities happening during diagnosis and intervention were occurring simultaneously. Furthermore, the median time from birth to intervention, and the median time from birth to confirmation of diagnosis, were both more than two times faster for CAH cases than non-CAH cases. It should be noted that all non-CAH cases are inclusive of time-critical and non-time-critical disorders, and have differing denominators based on data entered in the NewSTEPs data repository. This is consistent with state profile data findings, suggesting that NBS programs may prioritize running tests and reporting results for time-critical disorders. 

The analysis of the CAH case data submitted by 13 NBS programs for all three years shows that the median time interval of birth to the confirmation of diagnosis was the same compared to that of all CAH cases entered. This indicates that there is not a significant difference in time to diagnosis between the 13 programs that entered data for all three years versus programs did that not enter diagnosis information for all three years.

## 5. Conclusions

US NBS programs differ in hours of operation, as well as in follow-up definitions and procedures, however the screening methodologies and equipment used for CAH NBS are similar across the US. Furthermore, while there is variability in NBS systems across the US, NewSTEPs facilitates harmonization, and coordinates efforts amongst programs through national data collection, data analysis and technical assistance in order to improve the NBS system. 

NewSTEPs’ public health surveillance case definitions allow for consistent categorization of newborns at risk of developing an NBS condition. However, there is a lack of data being entered for the diagnostic workup section in the NewSTEPs data repository, making it difficult to apply NewSTEPs case definitions to data. For those programs that have entered data, it is often incomplete, with missing fields. There is a need for better quality CAH-confirmed case data. NewSTEPs continues to encourage NBS programs to provide more complete data, in order to be able to ask questions of datasets and accurately answer those questions.

Although there is room for improvement, the 35 contributing NBS programs fall within the national timeliness recommendations for specimen collection and the reporting of out-of-range results for CAH as a time-critical disorder. This is vital to ensuring that newborns at risk of CAH are appropriately seen by a medical provider in a timely manner. NewSTEPs supports NBS programs in improving timeliness by providing essential tools and techniques needed to successfully implement quality improvement initiatives and champion a quality improvement culture. These tools include national webinars archived on the NewSTEPs’ comprehensive resource center, data support and visualization to track improvement, funding opportunities to support initiatives, as well as online forums, training and customized coaching. NewSTEPs will continue to give feedback and data back to NBS programs, and support them in being more analytical towards their screening outcomes. This NewSTEPs model can serve as an example for NBS quality improvement practices worldwide.

Despite data entry challenges, NewSTEPs is committed to increasing the quantity and quality of case definition data in the repository, and will continue to do so via targeted outreach and pursuit of automated data entry options. It is vital for NBS programs to close the case loop with providers and get data back at the public health level, so as to adequately track and monitor newborns at the local, regional and national levels.

## Supplementary Materials

Supplementary materials can be found at https://www.mdpi.com/2409-515X/6/3/64/s1.
