# Supplementary material for: Landscape of Congenital Adrenal Hyperplasia Newborn Screening in the United States
Source: Int J Neonatal Screen. 2020 Aug 14;6(3):64. doi: 10.3390/ijns6030064 (PMC7569894; doi:10.3390/ijns6030064)
Supplement: Supplementary file 1 [file IJNS-06-00064-s001.pdf]

Article

Landscape of Congenital Adrenal Hyperplasia newborn screening in the United States

Sari Edelman [1*], Hiral Desai [1], Trey Pigg [1], Careema Yusuf [1] and Jelili Ojodu [1]

[1] Association of Public Health Laboratories, Silver Spring, MD 20910, USA

Supplementary Materials

NewSTEPs Public Health Surveillance Case Definitions:

21-Hydroxylase Deficiency – Classic Salt Wasting

Category	Serum 17- OHP - baseline or ACTH stimulated*	Urinary steroid profiling	Serum Sodium mE q/L	Plasma Renin Activity	CYP21A2 Mutation Analysis	If available - Supportive Clinical or Laboratory Evidence
Definite	> 10,000	Untested or unknown	< 135	Untested or unknown	Untested or unknown	Evidence of salt wasting (present in shock or severe failure to thrive)
Definite	> 10,000	Untested or unknown	<135	Untested or unknown	Untested or unknown	ambiguous genitalia in 46, XX
Definite	> 10,000	Untested or unknown	<135	Untested or unknown	Untested or unknown	other hormonal evidence of CAH
Definite	> 10,000	Untested or unknown	Untested or unknown	Elevated for age	Untested or unknown	Evidence of salt wasting (present in shock or severe failure to thrive)

Definite	> 10,000	Untested or unknown	Untested or unknown	Elevated for age	Untested or unknown	ambiguous genitalia in 46, XX
Definite	> 10,000	Untested or unknown	Untested or unknown	Elevated for age	Untested or unknown	other hormonal evidence of CAH
Definite	Untested or unknown	Untested or unknown	Untested or unknown	Untested or unknown	two classic gene mutations or deletions *in trans*	Evidence of salt wasting (present in shock or severe failure to thrive)
Definite	Untested or unknown	Untested or unknown	Untested or unknown	Untested or unknown	two classic gene mutations or deletions *in trans*	ambiguous genitalia in 46, XX
Definite	Untested or unknown	Untested or unknown	Untested or unknown	Untested or unknown	two classic gene mutations or deletions *in trans*	other hormonal evidence of CAH
Definite	Untested or unknown	(mass spectrometry) indicative of 21-Hydroxylase Deficiency CAH	Untested or unknown	Untested or unknown	Untested or unknown	Evidence of salt wasting (present in shock or severe failure to thrive)
Definite	Untested or unknown	(mass spectrometry) indicative of 21-Hydroxylase Deficiency CAH	Untested or unknown	Untested or unknown	Untested or unknown	ambiguous genitalia in 46, XX

21-Hydroxylase Deficiency – Classic Salt Wasting

Category	Serum 17-OHP - baseline or ACTH stimulated*	Urinary steroid profiling	Serum Sodium mEq/ L	Plasma Renin Activity	CYP21A2 Mutation Analysis	If available - Supportive Clinical or Laboratory Evidence
Definite	Untested or unknown	(mass spectrometry) indicative of 21-Hydroxylase Deficiency CAH	Untested or unknown	Untested or unknown	Untested or unknown	other hormonal evidence of CAH
Probable	1,000 -10,000	Untested or unknown	< 135	Untested or unknown	Untested or unknown	Evidence of salt wasting (present in shock or severe failure to thrive)
Probable	1,000 -10,000	Untested or unknown	< 135	Untested or unknown	Untested or unknown	ambiguous genitalia in 46,XX
Probable	1,000 -10,000	Untested or unknown	< 135	Untested or unknown	Untested or unknown	other hormonal evidence of CAH
Probable	1,000 -10,000	Untested or unknown	Untested or unknown	Elevated for age	Untested or unknown	Evidence of salt wasting (present in shock or severe failure to thrive)

Possible	1,000 -10,000	Untested or unknown	Untested or unknown	Elevated for age	Untested or unknown	ambiguous genitalia in 46,XX
Possible	1,000 -10,000	Untested or unknown	Untested or unknown	Elevated for age	Untested or unknown	other hormonal evidence of CAH

21-Hydroxylase Deficiency-Classical Simple Virilizing

Category	Serum 17-OHP - baseline or ACTH stimulated*	Urinary Steroid profiling	Serum Sodium mEq/L	Plasma Renin Activity	CYP21 A2 Mutation Analysis	If available - Supportive Clinical or Laboratory Evidence
Definite	>10,000	Untested or unknown	>135	Untested or unknown	Untested or unknown	Ambiguous genitalia in 46,XX
Definite	>10,000	Untested or unknown	>135	Untested or unknown	Untested or unknown	no evidence of salt wasting
Definite	>10,000	Untested or unknown	>135	Untested or unknown	Untested or unknown	other hormonal evidence of CAH
Definite	>10,000	Untested or unknown	Untested or unknown	Normal for age	Untested or unknown	Ambiguous genitalia in 46,XX
Definite	>10,000	Untested or unknown	Untested or unknown	Normal for age	Untested or unknown	no evidence of salt wasting
Definite	>10,000	Untested or unknown	Untested or unknown	Normal for age	Untested or unknown	other hormonal evidence of CAH
Definite	Untested or unknown	(mass spectrometry) indicative of 21-Hydroxylase Deficiency CAH	>135	Untested or unknown	Untested or unknown	Ambiguous genitalia in 46,XX

Definite	Untested or unknown	(mass spectrometry) indicative of 21-Hydroxylase Deficiency CAH	>135	Untested or unknown	Untested or unknown	no evidence of salt wasting
Definite	Untested or unknown	(mass spectrometry) indicative of 21-Hydroxylase Deficiency CAH	>135	Untested or unknown	Untested or unknown	other hormonal evidence of CAH
Definite	Untested or unknown	(mass spectrometry) indicative of 21-Hydroxylase Deficiency CAH	Untested or unknown	Normal for age	Untested or unknown	Ambiguous genitalia in 46,XX

21-Hydroxylase Deficiency-Classical Simple Virilizing

Category	Serum 17-OHP - baseline or ACTH stimulated*	Urinary Steroid profiling	Serum Sodium mEq/L	Plasma Renin Activity	CYP21A2 Mutation Analysis	If available - Supportive Clinical or Laboratory Evidence
Definite	Untested or unknown	(mass spectrometry) indicative of 21-Hydroxylase Deficiency CAH	Untested or unknown	Normal for age	Untested or unknown	no evidence of salt wasting
Definite	Untested or unknown	(mass spectrometry) indicative of 21-Hydroxylase Deficiency CAH	Untested or unknown	Normal for age	Untested or unknown	other hormonal evidence of CAH
Definite	Untested or unknown	Untested or unknown	>135	Untested or unknown	two classic gene mutations or deletions *in trans*	Ambiguous genitalia in 46,XX
Definite	Untested or unknown	Untested or unknown	>135	Untested or unknown	two classic gene mutations or deletions *in trans*	no evidence of salt wasting
Definite	Untested or unknown	Untested or unknown	>135	Untested or unknown	two classic gene mutations or deletions *in trans*	other hormonal evidence of CAH
Definite	Untested or unknown	Untested or unknown	Untested or unknown	Normal for age	two classic gene mutations or deletions *in trans*	Ambiguous genitalia in 46,XX

Definite	Untested or unknown	Untested or unknown	Untested or unknown	Normal for age	two classic gene mutations or deletions *in trans*	no evidence of salt wasting
Definite	Untested or unknown	Untested or unknown	Untested or unknown	Normal for age	two classic gene mutations or deletions *in trans*	other hormonal evidence of CAH
Probable	1,000 -10,000	Untested or unknown	>135	Untested or unknown	Untested or unknown	Ambiguous genitalia in 46,XX or normal genitalia in 46,XY
Probable	1,000 -10,000	Untested or unknown	Untested or unknown	Normal for age	Untested or unknown	Ambiguous genitalia in 46,XX or normal genitalia in 46,XY
Probable	1,000 -10,000	Untested or unknown	Untested or unknown	Untested or unknown	Untested or unknown	no evidence of salt wasting
